# Linking the Weibull distribution to Gini coefficients: a bamboo specific framework for intra-culm leaf area inequality

**DOI:** 10.3389/fpls.2025.1685552

**Published:** 2025-12-18

**Authors:** Zhifei Jiao, Shuai Liu, Karl J. Niklas, Christian Frølund Damgaard, Weihao Yao, Meng Lian, Feixue Jiang, Peijian Shi

**Affiliations:** 1 Bamboo Research Institute, Nanjing Forestry University, Nanjing, China; 2 College of Ecology and Environment, Nanjing Forestry University, Nanjing, China; 3 School of Integrative Plant Science, Cornell University, Ithaca, NY, United States; 4 Department of Ecoscience, Aarhus University, Aarhus, Denmark

**Keywords:** distribution function, Gini coefficient, leaf area, Lorenz curve, maximum likelihood method, scale parameter

## Abstract

Quantifying inequality in the leaf area distribution within a single module is critical for elucidating plant resource allocation strategies, but the accuracy of theoretical Gini coefficients derived from statistical distributions remains poorly validated against observed values. To resolve this gap, we analyzed 9,242 leaves from 121 culms of the bamboo *Semiarundinaria densiflora*, a model system with minimal ontogenetic noise and moderate leaf counts (36–187 leaves per culm) that enables robust Lorenz curve construction. Four candidate distributions were tested: the normal, log-normal, two-parameter Gamma, and two-parameter Weibull distributions. The parameters of the normal and log-normal distributions were estimated directly from sample statistics, whereas the parameters of the Gamma and Weibull distributions were estimated using the maximum likelihood method. Goodness of fit was assessed using the Kolmogorov-Smirnov (K-S) test for distributional validity, and the Akaike’s information criterion (AIC) for model selection. Although the Gamma distribution passed the K-S test for a slightly higher percentage of culms (99%) than the Weibull distribution (97.5%), the Weibull distribution was selected as the superior model because it yielded significantly lower AIC values. Crucially, the theoretical Gini coefficients of the Gamma and Weibull distributions (denoted as *G_G_* and *G_W_*, respectively) were tested against the observed Gini coefficients (*G_P_*) calculated nonparametrically using the polygon method. Linear regression demonstrated that *G_W_*​ predicted *G_P_* with near isometric accuracy: the intercept’s 95% confidence interval included zero (−0.006 to 0.017) and the slope’s 95% confidence interval included unity (0.929 to 1.039). In contrast, *G_G_* exhibited significant bias. Notably, pooling leaves across culms violated all distributions due to microhabitat driven multimodality, confirming that intra-culm inequality assessments require organism level analysis. This work provides an empirical validation that the Weibull shape parameter reliably quantifies intra-culm leaf area inequality. By bridging theoretical distribution models with field-derived inequality metrics, our approach provides insights into canopy efficiency, photosynthetic optimization, and hydraulic trade-offs. Future work should test this approach in other grass species and assess its generalizability in plants with contrasting canopy architectures.

## 1 Introduction

The evolution of large leaves represents a pivotal innovation in vascular plant evolution, facilitating dramatic increases in photosynthetic capacity that enhanced terrestrial productivity (Niklas, 1997). As the primary interface for light capture, gas exchange, and thermoregulation, leaves exhibit remarkable morphological diversity shaped by natural selection across environmental gradients (Wright et al., 2004). In forest ecosystems, canopy structure and leaf area distribution indirectly govern light interception efficiency and carbon sequestration (Poorter et al., 2016). Similarly, urban trees leverage foliar photosynthetic capacity, which depends critically on both total leaf area and its distribution, to mitigate heat islands and absorb atmospheric pollutants (Nowak et al., 2006, 2008). Within this context, variation in leaf area at the intra-plant and intra-culm levels, which reflects strategic optimization of this key functional trait, mediates physiological performance, resource allocation trade-offs, and fitness outcomes (Westoby et al., 2002; Weiner and Thomas, 1986; Lian et al., 2024). Larger leaves enhance light harvesting but incur higher construction costs, self-shading, and hydraulic risks, whereas smaller leaves reduce self-shading and confer thermodynamic advantages in harsh climates (Milla and Reich, 2007; Baird et al., 2021). Consequently, variation reflects strategic optimization of carbon investment under developmental and environmental constraints (Lian et al., 2023). Moreover, the distribution of leaf area within a single module serves as a quantifiable signature of adaptive plasticity, enhancing competitive ability in dense stands and resilience to abiotic stressors (Schmitt and Boisseaux, 2023). Understanding these principles is fundamental to predicting plant responses to global change drivers such as intensified droughts or elevated CO_2_ (Poorter et al., 2009; Swann et al., 2016; Hu et al., 2025).

Quantifying inequality in leaf area distribution within a single module is critical for understanding plant photosynthetic optimization strategies, as heterogeneity along a single stem reflects micro-environmental gradients and resource allocation trade-offs. Traditional metrics, such as the coefficient of variation (CV), measure dispersion but disregard distribution shape (Bendel et al., 1989), whereas the Gini coefficient (GC) derived from Lorenz curves holistically captures “size hierarchies” defined by high variability and a skewed structure where few large leaves dominate resources over many small ones (Weiner and Solbrig, 1984; Lian et al., 2024). Statistically, biological size distributions often follow right-skewed forms, with Gamma and Weibull families being prominent due to their flexibility and their close mathematical relationship to the exponential distribution. The Gamma distribution models additive processes as sums of exponential variables, fitting scenarios like cumulative growth increments in tree diameters (Zhang et al., 2003), whereas the Weibull distribution characterizes extreme values of exponential samples, capturing multiplicative effects or size dependent mortality in leaf reliability frameworks (Weibull, 1951; Taubert et al., 2013). However, prior applications have focused overwhelmingly on variation in leaf area at the inter-plant and inter-culm levels, such as population-level leaf sampling (Shi et al., 2019) or stand level tree diameters (Zhang et al., 2003), neglecting intra-culm distributions where leaf area inequality directly governs photosynthetic efficiency. Critically, for standard two-parameter distributions like Gamma and Weibull (with minimum size fixed at zero), the GC exhibits consistent positive covariation with skewness (Lian et al., 2024). This tight mathematical coupling between distribution parameters and the GC indicates that scale-dependent effects may mask true biological inequality in intra-culm leaf area distributions, necessitating distribution-specific calibration of GC to resolve how intrinsic allometry and resource partitioning shape canopy efficiency.

**Figure 1:**
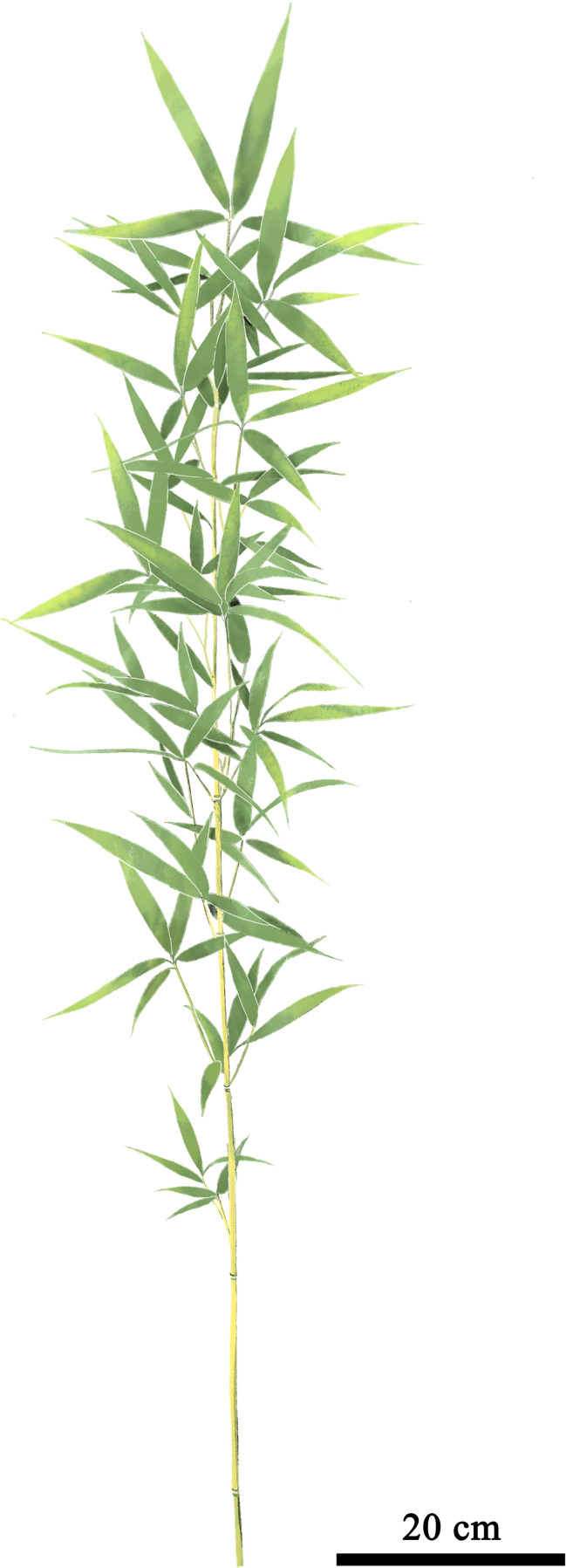
Free-hand drawing of the above-ground part of *Semiarundinaria densiflora* (Rendle) T. H. Wen.

Bamboos (Poaceae: Bambusoideae), comprising over 1,600 species, present an ideal model system for investigating intra-culm inequality in leaf area distribution (Clark et al., 2015). Their culm-specific (per-stem) leaf cohorts exhibit minimal ontogenetic variation compared to broadleaved species, reducing age-related noise in trait distributions (Shi et al., 2015, 2021). Shrub bamboos, like *Semiarundinaria densiflora* (Rendle) T. H. Wen (a mixed-rotating bamboo with a near monopodial growth habit), feature moderate leaf counts per culm (typically 30–200 leaves per shoot) that are sufficiently large yet tractable numbers for exhaustive measurement suitable for robust Lorenz curve construction (**Figure 1**). Such sizes avoid the undersampling biases plaguing large-statured bamboos (e.g., *Phyllostachys edulis* (Carrière) J. Houzeau with 400–800 leaves per culm), while exceeding the limited sample sizes of smaller dwarf bamboos (e.g., *Shibataea chinensis* Nakai with 10−40 leaves and *Sasaella kongosanensis* ‘Aureostriatus’ with only 1−19 leaves; Wang et al., 2024). Critically, dense foliage in bamboos drives intense intra-culm competition for light, potentially amplifying leaf area asymmetry (Wang et al., 2018; Huang et al., 2023). Here, we test whether intra-culm leaf area distributions in *S. densiflora* conform to the two-parameter Gamma distribution or the two-parameter Weibull distribution, and evaluate if their theoretical GCs accurately reflect the observed GCs calculated nonparametrically using the polygon method (i.e., based on the Lorenz curve). Specifically, we ask: (i) do the Gamma and Weibull distributions adequately describe intra-culm leaf area variation? (ii) which distribution provides superior fit? and (iii) can theoretical GCs (calculated from distribution parameters) be equivalent to the observed polygonal GCs? By integrating distributional modeling with inequality metrics, this work sets up a baseline for describing phenotypic variation in leaf size within and across individual culms (or plants).

## 2 Materials and methods

### 2.1 Leaf sampling

A total of 121 culms of *Semiarundinaria densiflora* were sampled in October 2024 from the Whitehorse Experimental Station of Nanjing Forestry University, Nanjing, China (119°09′14″ E, 31°36′48″ N). The species, originally introduced from the Nanjing Forestry University Xinzhuang Campus in 2013, has naturalized at the sampling site. Each culm’s aboveground portion (**Figure 1**) was excised at ground level, immediately wrapped in moist paper, and transported to the laboratory within two hours to reduce the effects of dehydration.

### 2.2 Data acquisition

Leaves were excised from culms and their pseudo-petioles removed. Individual leaves were scanned at 600 dpi resolution using a photo scanner (Epson V550, Batam, Indonesia) and saved as .jpg files. Images were cropped, converted to black–white format using Adobe Photoshop 2021 (version 22.4.2; Adobe, San Jose, CA, USA), and saved as .bmp files. Planar boundary coordinates of each leaf were extracted using a custom MATLAB function (MATLAB ≥ R2009a; MathWorks, Natick, MA, USA) developed by Su et al. (2019). Lamina area (*A*) of each of the 9,242 leaves calculated using the “bilat” function in the “biogeom” package (version 1.3.6; Shi et al., 2022) implemented in R (version 4.3.1; R Core Team, 2023). Leaf lamina area data are assessable online from **Supplementary Table S1**.

### 2.3 Distribution functions

Four probability density functions (i.e., the normal, log-normal, two-parameter Gamma and two-parameter Weibull distributions, i.e., Equations 1–4) were used to describe the individual leaf area distribution for each of the 121 the culms.

The normal distribution function $f_{N}\left(x\right)$ takes the form:

(1) $$f_{N}\left(x\right)=\frac{1}{\sqrt{2\pi }\sigma }\text{exp}\left[-\frac{{\left(x-\mu \right)}^{2}}{2\sigma^{2}}\right]$$

where *x* represents individual leaf area; *μ* and *σ* represent the mean and the standard deviation of the leaf areas per culm, respectively.

The log-normal distribution function $f_{L}\left(x\right)$ takes the form

(2) $$f_{L}\left(x\right)=\frac{1}{\sqrt{2\pi }\sigma_{\text{log}}x}\text{exp}\left\{-\frac{{\left\{\log\left(x\right)-\mu_{\text{log}}\right\}}^{2}}{2\sigma_{\text{log}}^{2}}\right\}$$

where *μ*_log_ and *σ*_log_ represent the mean and the standard deviation of the log-transformed leaf areas; *x* > 0.

The two-parameter Gamma distribution function $f_{G}\left(x\right)$ takes the form

(3) $$f_{G}\left(x\right)=\frac{1}{b^{k}\text{ Γ}\left(k\right)}x^{k-1}\text{exp}\left(-\frac{x}{b}\right)$$

where *k* and *b* represent the shape parameter and scale parameter that are both greater than zero, respectively; *x* ≥ 0; Γ(*k*) is the gamma function, which equals $\int_{0}^{\infty }t^{k-1}e^{-t}dt$.

The two-parameter Weibull distribution function $f_{W}\left(x\right)$ takes the form

(4) $$f_{W}\left(x\right)=\frac{\alpha }{\beta }{\left(\frac{x}{\beta }\right)}^{\alpha -1}\text{exp}\left[-{\left(\frac{x}{\beta }\right)}^{\alpha }\right]$$

where *α* and *β* represent the shape parameter and scale parameter; *x* > 0. There are three cases of the numerical value of *α*: (i) *α <* 3.6, a right-skewed distribution is indicated; (ii) *α* > 3.6, a left-skewed distribution is indicated; (iii) *α* = 3.6, a symmetrical distribution is indicated (Murthy et al., 2004).

### 2.4 Parameter estimation and statistical test of significance of the distributions

Parameters for the normal and log-normal distributions were estimated directly from sample statistics: the mean ( $\hat{\mu }=\bar{x}$) and standard deviation ( $\hat{\sigma }=s_{x}$) of raw leaf areas per culm defined the normal distribution; the mean ( ${\hat{\mu }}_{\text{log}}=\bar{\log\left(x\right)}$) and standard deviation ( ${\hat{\sigma }}_{\text{log}}=s_{\text{log}\left(x\right)}$) of log-transformed data per culm characterized the log-normal distribution. For the two-parameter Gamma and Weibull distributions, parameters were estimated by means of the maximum likelihood method for each culm using the “mle2” function in the “bbmle” package (version 1.0.25.1; Bolker, 2008) under R (version 4.3.1; R Core Team, 2023). Akaike’s information criterion (AIC) values were computed for each culm to estimate the relative information loss of candidate distributions, thereby enabling model comparison based on trade-offs between goodness-of-fit and parameter complexity (Spiess and Neumeyer, 2010). Paired *t*-tests were used to test the statistical significance of the difference in the AIC values between the two-parameter Gamma and Weibull distributions.

Distribution validity was evaluated through two distinct statistical tests. First, the Shapiro-Wilk test (Royston, 1995) assessed normality and log-normality using raw and log-transformed leaf area data, respectively. Second, the Kolmogorov-Smirnov (K-S) test (Schröer and Trenkler, 1995) specifically quantified goodness-of-fit for the two-parameter Gamma and Weibull distributions by comparing the empirical cumulative distribution function with the theoretical cumulative distribution function.

### 2.5 Calculation of Gini coefficients

**Figure 2:**
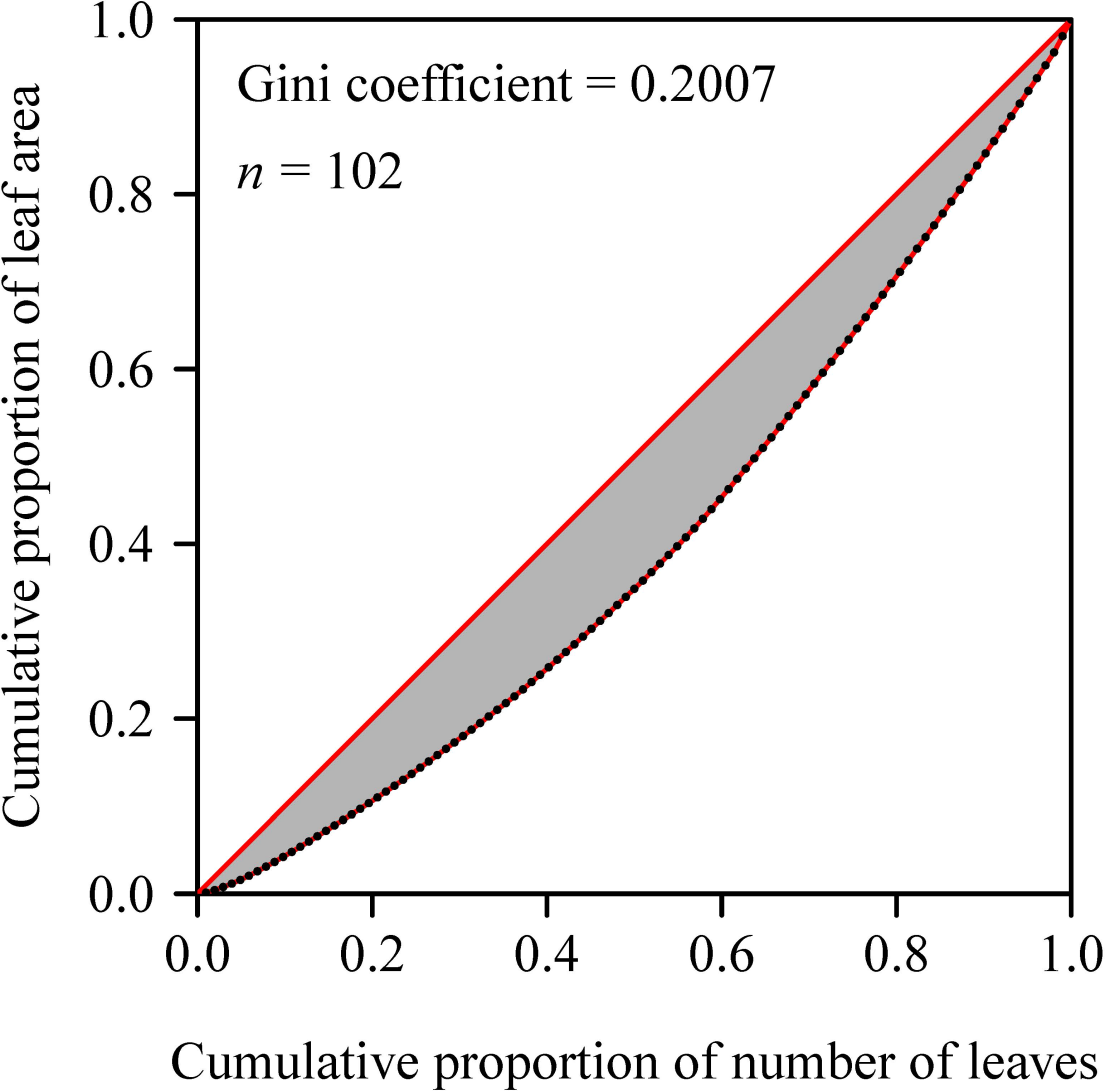
The Lorenz curve formed by the accumulative proportion of leaf area per culm plotted against the accumulative proportion of the number of leaves per culm. The Gini coefficient is defined as twice the area of the shaded region bounded by the line of absolute equality and the Lorenz curve.

The Gini coefficient (GC; Gini, 1912) is defined based on the Lorenz curve (Lorenz, 1905), which plots the accumulative proportion of leaf area per culm against the accumulative proportion of the number of leaves per culm (**Figure 2**). The GC equals twice the area enclosed by the Lorenz curve and the line of absolute equality. As a rule of thumb, when the total number of leaves per culm is large (≥ 30), the polygon method can accurately calculate the GC. Given the number of leaves per culm of *S. densiflora* ranging between 36 and 187 with a mean 76 in the 121 sampled culms, the GC calculated using the polygon method can be regarded as the ‘observed’ GC, which is denoted as *G_P_* for convenience hereinafter.

The Lorenz curve can be defined by the quantile function of *F*(*x*), where *F*(*x*) is the cumulative distribution function, and *x* in the context of this study represents the individual leaf area. The Lorenz function, *L*(*p*), where *p* represents the cumulative proportion of the number of leaves (sorted by the ascending leaf area sequence) ranging between 0 and 1, takes the form (Gastwirth, 1971; Damgaard and Weiner, 2000; Lian et al., 2024):

(5) $$L\left(p\right)=\mu^{-1}\int_{0}^{p}F^{-1}\left(q\right)dq$$

where *μ* represent the population mean of leaf area, equaling $\int_{0}^{\infty }xf\left(x\right)dx$; *q* is the quantile ranging between 0 and 1, and $F^{-1}\left(q\right)$ is the quantile function of *F*(*x*). Consequently, based on Equation 5, the Gini coefficient (*G*) can be derived as (Bendel et al., 1989; Lian et al., 2024):

(6) $$G=1-2\int_{0}^{1}L\left(p\right)dp=1-2\mu^{-1}\int_{0}^{1}\left(\int_{0}^{p}F^{-1}\left(q\right)dq\right)dp$$

Based on Equation 6, it is easy to derive the theoretical Gini coefficients (see Equations 7–10) of the foregoing four distribution functions, i.e., Equations 1–4 (Bendel et al., 1989; Lian et al., 2024). Let *G_N_*, *G_L_*, *G_G_* and *G_W_* represent the theoretical Gini coefficients derived from the normal, lognormal, two-parameter Gamma, and two-parameter Weibull distributions, respectively. It therefore follows that (Bendel et al., 1989):

(7) $$G_{N}=\frac{\sigma }{\mu \sqrt{\pi }}$$

where *μ* and *σ* represent the mean and the standard deviation of the leaf areas per culm, respectively;

(8) $$G_{L}=2\text{Φ}\left(\frac{\sigma_{\text{log}}}{\sqrt{2}}\right)-1$$

where *σ*_log_ represents the standard deviation of the log-transferred leaf areas per culm; Φ(·) is the cumulative distribution function of the standard normal distribution;

(9) $$G_{G}=\frac{\text{Γ}\left(k+0.5\right)}{\sqrt{\pi }\text{ Γ}\left(k+1\right)}$$

where *k* the shape parameter of the two-parameter Gamma distribution; and

(10) $$G_{W}=1-2^{-\frac{1}{\alpha }}$$

where *α* is the shape parameter of the two-parameter Weibull distribution.

We used the estimated parameters of the two-parameter Gamma and two-parameter Weibull distributions based on the maximum likelihood method to calculate *G_G_* and *G_W_*.

For the distributions that passed the statistical tests of significance (in the case of most culms), the linear regression between the theoretical and observed Gini coefficients (e.g., *G_W_* versus *G_P_*) was carried out to test whether there was an isometric relationship. In theory, the intercept equals 0 and the slope equals unity. Reduced major axis protocols (Niklas, 1994) were used to estimate the intercept and slope, and the bootstrap percentile method (Efron and Tibshirani, 1993; Sandhu et al., 2011) was used to calculate the 95% confidence intervals (CIs) of the intercept and slope.

## 3 Results

**Figure 3:**
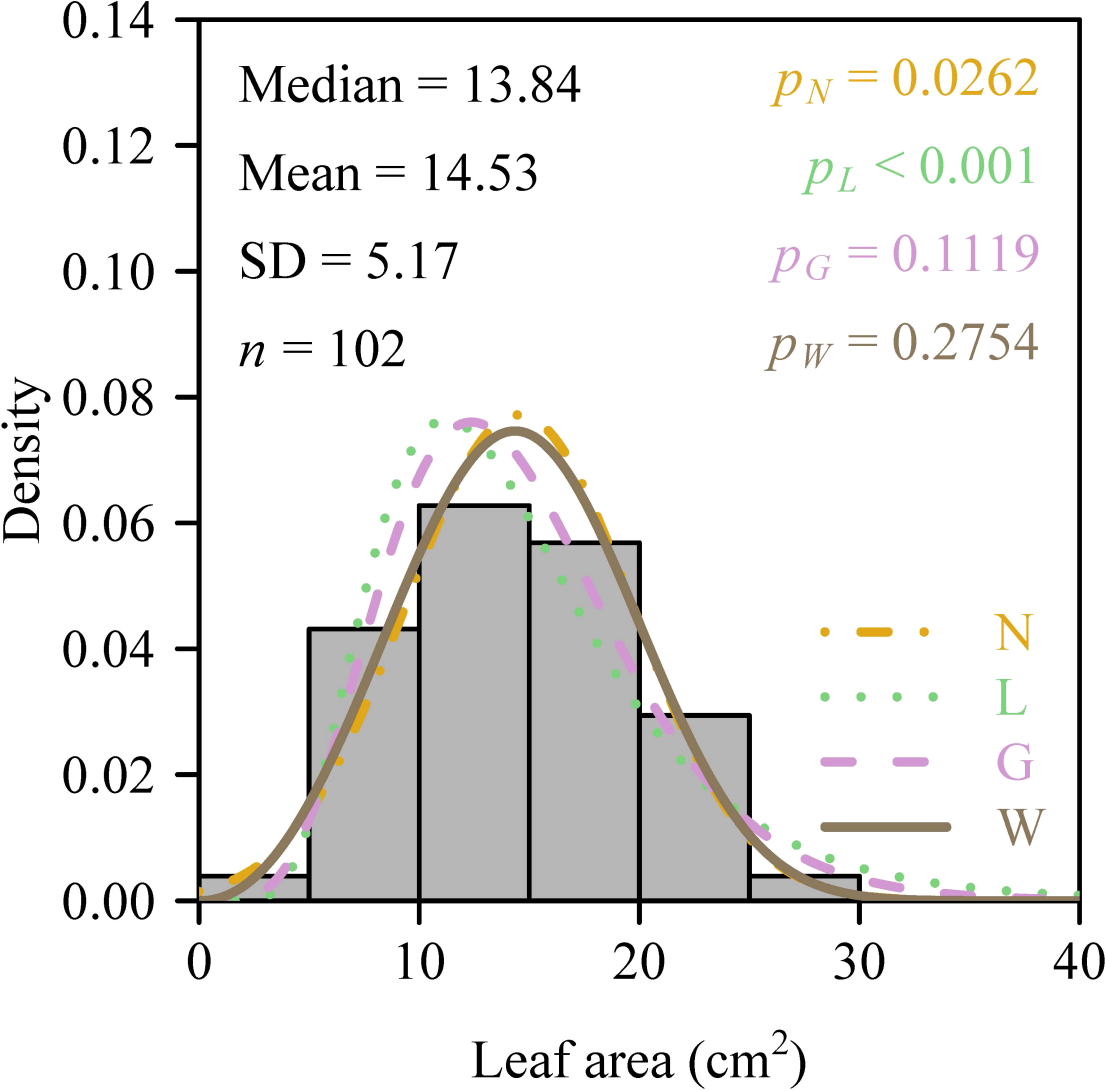
Individual leaf area distribution of a representative *Semiarundinaria densiflora* culm. “Mean” and “Median” are the mean and median, respectively; “SD” is the standard deviation; *n* is the number of leaves on the culm; *p_N_* is the probability that the data are consistent with the null hypothesis of a normal distribution; *p_L_* is the probability that the data are consistent with the null hypothesis of a log-normal distribution; *p_G_* is the probability that the data are consistent with the null hypothesis of the two-parameter Gamma distribution; *p_W_* is the probability that the data are consistent with the null hypothesis of the two-parameter Weibull distribution. The colorful curves represent the predicted probability densities for the four distribution functions. “N”, “L”, “G” and “W” for different types of lines represent the norm, log-normal, Gamma and Weibull distributions, respectively.

**Figure 4:**
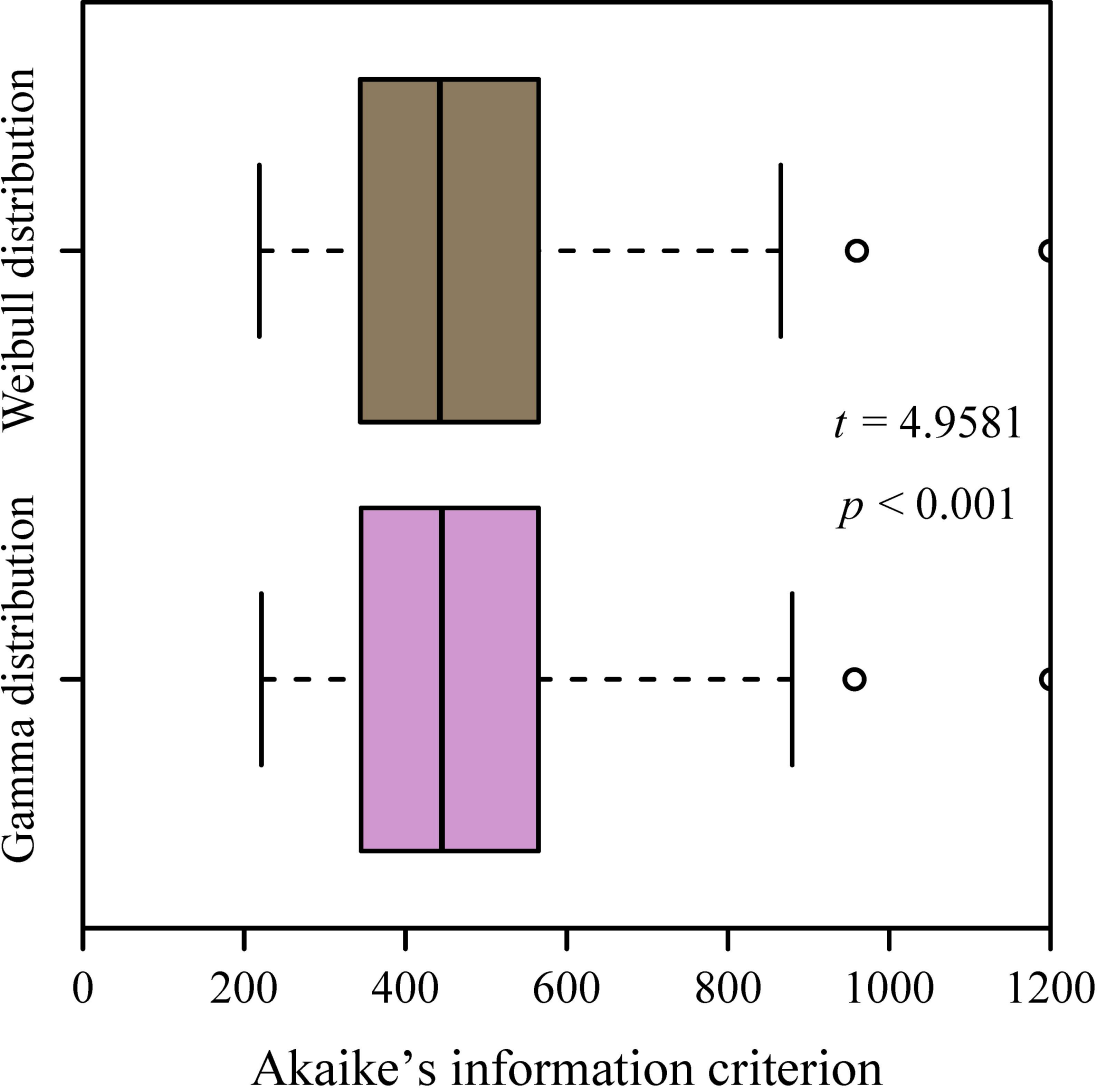
Comparison of the Akaike’s information criterion (AIC) values of fitting the Gamma and Weibull distributions. The paired *t*-test was used to test the significance of the difference in the AIC values between the two distributions.

**Figure 5:**
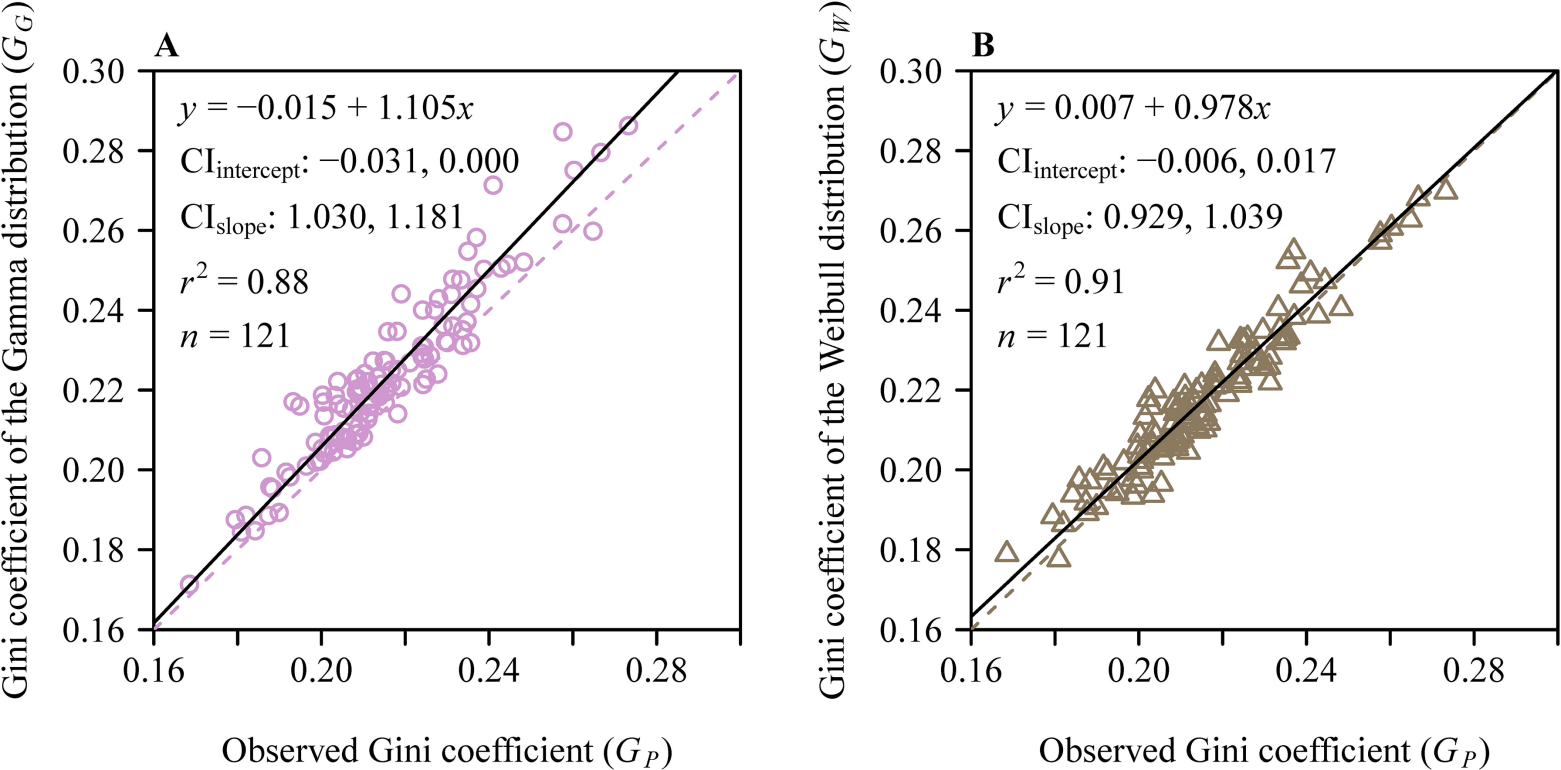
Linear fits to **(A)** the theoretical Gini coefficient of the Gamma distribution (*G_G_*) versus the observed Gini coefficient calculated using the polygon method (*G_P_*), and **(B)** the theoretical Gini coefficient of the Weibull distribution (*G_W_*) versus *G_P_*. For each panel, *y* represents *G_G_* in panel **(A)** or *G_W_* in panel **(B)**, and *x* represents *G_P_*; the CI_intercept_ is the 95% confidence interval of the intercept; the CI_slope_ is the 95% confidence interval of the slope; *r*^2^ is the coefficient of determination; *n* is the sample size, i.e., the number of bamboo culms.

**Figure 6:**
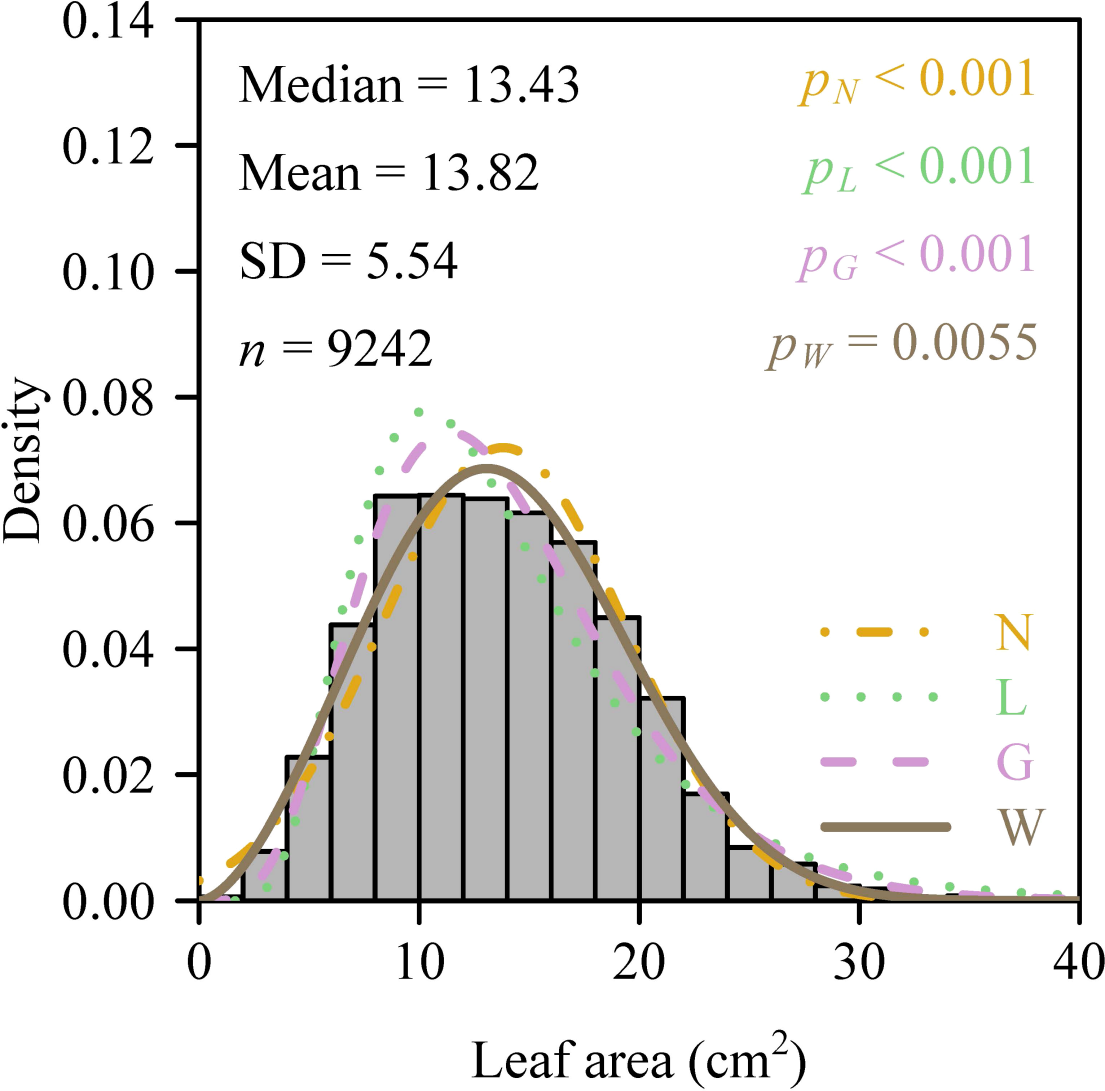
Individual leaf area distribution of the 121 *Semiarundinaria densiflora* culms. “Mean” and “Median” are the mean and median, respectively; “SD” is the standard deviation; *n* is the total number of leaves in the 121 culms; *p_N_* is the probability that the data are consistent with the null hypothesis of a normal distribution; *p_L_* is the probability that the data are consistent with the null hypothesis of a log-normal distribution; *p_G_* is the probability that the data are consistent with the null hypothesis of the two-parameter Gamma distribution; *p_W_* is the probability that the data are consistent with the null hypothesis of the two-parameter Weibull distribution. The colorful curves represent the predicted probability densities for the four distribution functions. “N”, “L”, “G” and “W” for different types of lines represent the norm, log-normal, Gamma and Weibull distributions, respectively.

The mean aboveground height of the 121 sampled culms was 116.7 ± 20.2 cm (mean ± SD), and the mean number of leaves per culm was 76 ± 25 (range: 36–187). Analysis of intra-culm leaf area distribution showed that 56.2% of culms passed the normality test, whereas 19.8% satisfied log-normality. Critically, 99% of culms (120 out of 121) conformed to the two-parameter Gamma distribution, and 97.5% (118 culms) fit the two-parameter Weibull distribution (**Figure 3**). All Weibull shape parameters (2.20−3.55) fell below 3.6, confirming consistent right-skewed distributions. Paired *t*-tests showed significantly lower Akaike’s Information Criterion (AIC) values for the Weibull distribution compared to the Gamma distribution (*p* < 0.05; **Figure 4**). When comparing theoretical and observed Gini coefficients, the regression of the theoretical Gini coefficient of the Gamma distribution (*G_G_*​) against the observed Gini coefficient calculated using the polygon method (*G_P_*​)​ yielded a slope whose 95% CI lower bound exceeded unity, indicating an allometry. Conversely, for the theoretical Gini coefficient of the Weibull distribution (*G_W_*​) versus *G_P_*​, the intercept’s 95% CI (−0.006 to 0.017) included zero and the slope’s 95% CI (0.929 to 1.039) included unity (**Figure 5**), demonstrating optimal characterization of intra-culm inequality in leaf area distribution by the two-parameter Weibull distribution. However, the combined leaf area distribution across all 121 culms violated all four tested distributions (*p* < 0.05; **Figure 6**). The failure to fit any of the candidate distributions at the pooled level illustrates a key methodological insight: aggregating data across all individual culms masks the consistent intra-culm signals, thereby justifying our focus on the individual culm level to assess intrinsic leaf area inequality.

## 4 Discussion

This study confirms the two-parameter Weibull distribution as a robust model for quantifying intra-culm leaf area inequality in *Semiarundinaria densiflora*, as validated by stringent statistical comparisons of theoretical and observed Gini coefficients. In the following sections, we contextualize the biological implications of right-skewed leaf size distributions, evaluate methodological advances in inequality quantification, and address limitations and directions for future research.

### 4.1 Biological drivers of right-skewed leaf area distributions

Here, we argue that the consistent right-skewed leaf area distributions across *S. densiflora* culms, characterized by Weibull shape parameters (*α*) ranging from 2.20 to 3.55 (mean = 2.85), reflect adaptive optimization under vertical light gradients. Apical dominance mechanisms have been argued to favor resource allocation to upper, acropetally located leaves, resulting in larger lamina areas positioned higher on the culm to maximize photosynthetic gain under direct irradiance (Hirose and Werger, 1995; Anten and Hirose, 1999). This comes at the cost of smaller basal leaves, which are argued to minimize self-shading among lower branches, reducing metabolic costs while still contributing to carbon fixation (Falster and Westoby, 2003). In mixed stands with the shorter bamboo *Pleioblastus pygmaeus* (height: 5−95 cm), *S. densiflora*’s taller stature and the skewness of its leaf size distribution likely represents a competitive strategy to overtop neighbors and maximize light interception. Larger and higher leaves project beyond competing canopies, whereas a minimized basal foliage reduces respiratory load without compromising light interception potential, which aligns with the trade-off between photon capture efficiency and biomass investment observed in stratified plant communities (Hirose and Werger, 1995). The absence of a minimum leaf size threshold (location parameter *c* ≈ 0) likely indicates minimal developmental constraints on leaf area, unlike woody plants where hydraulic limitations impose size-related trade-offs (Ryan and Yoder, 1997). Smaller basal leaves may further confer hydraulic advantages during drought by reducing transpirational demands and xylem conduit tension in lower canopy regions, thereby reducing embolism risk and representing a mechanism analogous to size-structured foliage in tree canopies mitigating hydraulic vulnerability (Brodribb et al., 2020).

The growth form of *S. densiflora* as a mixed-rotating bamboo further sheds light on these findings. Its tendency toward a running (monopodial) habit promotes physiological modular independence among culms, making them suitable replicate units for studying intra-culm inequality. However, its simultaneous propensity to form dense stands creates a competitive light environment characterized by shading from neighboring culms of varying heights. The consistent right-skewed leaf area distribution observed within individual culms is likely an adaptive response to this intense intra-specific competition, driven by the stand structure that its mixed growth form produces. By allocating more resources to larger, apical leaves, a culm can effectively overtop its immediate neighbors, while reducing the size of basal leaves minimizes self-shading and construction costs. Thus, the observed inequality pattern can be interpreted as a culm-level strategy to optimize photosynthetic gain within the specific competitive microenvironment created by this bamboo’s unique growth architecture. In summary, each culm functions as an individual (modular) unit.

It is also worth noting that field observation during this study indicates that aggregated leaf areas across culms violate distribution models due to inter-culm microhabitat variation. Edge culms experience higher irradiance heterogeneity, promoting greater leaf size variability and skewness consistent with light-driven plasticity in grasses (Anten and Hirose, 1999), whereas shaded interior culms exhibit more uniform distributions. This microhabitat-driven divergence introduced multi-modality into pooled data, violating the assumption of unimodality for the two-parameter distributions. Consequently, intra-culm distributions remain well-described by Weibull models due to microenvironmental homogeneity along individual stems, whereas aggregated distributions reflect the compounding effects of inter-culm competition for light and spatial niche partitioning within the stand. The persistent right-skew within culms thus emerges as a quantifiable signature of adaptive architecture balancing photosynthetic gain, competitive dominance, and hydraulic safety.

The consistent superiority of the Weibull distribution over the Gamma distribution, as evidenced by lower AIC values, likely holds biological significance. The Weibull distribution is often used to model failure times or the distribution of extreme values in reliability engineering. In an ecological context, this can be interpreted as modeling the “risk” or “efficiency” of leaf area investment. The shape parameter (*α*) might reflect a plant’s strategy to optimize the trade-off between hydraulic safety (risk of embolism) and photosynthetic gain. A Weibull distribution can characterize a multiplicative growth process where the rate of leaf area expansion exhibits a form of “diminishing returns”, which is a pattern observed in plant allometry where increasing resource investment (e.g., in biomass) yields progressively smaller gains in output (e.g., leaf area or light capture efficiency) (Milla and Reich, 2007; Niklas et al., 2007). For example, in the case of bamboo leaves, the scaling of individual leaf area vs. leaf mass shows decreasing marginal returns, likely due to structural and hydraulic constraints that limit expansion efficiency as leaves grow larger (Huang et al., 2020; Guo et al., 2021). This pattern aligns with the Weibull distribution’s flexibility in capturing processes where growth decelerates due to increasing constraints, offering a more mechanistic description of carbon allocation strategy compared to the Gamma distribution, which often arises from the sum of independent exponential “waiting times” and may not as effectively capture such nonlinear scaling.

### 4.2 Methodological advances in inequality quantification

Our study bridges a critical gap by validating parametric Gini coefficients against empirical Lorenz curves. Previous assessments often relied solely on distribution models without empirical verification (Bendel et al., 1989; Sarabia, 1997; Huang et al., 2023; Sitthiyot and Holasut, 2023), risking biological misinterpretation. *Semiarundinaria densiflora*’s moderate leaf counts (36−187) enabled precise polygonal *G_P_* calculations, avoiding limitations in species with extreme leaf counts: large bamboos (e.g., *Phyllostachys edulis*) introduce measurement complexity, while small-statured species (e.g., *Shibataea chinensis* and *Sasaella kongosanensis* ‘Aureostriatus’) yield insufficient data for robust Lorenz curve construction. Sample sizes > 30 minimized nonlinearity-induced bias (Lian et al., 2024), ensuring statistical reliability. Although both Gamma and Weibull distributions showed high goodness-of-fit (K-S test: *p* > 0.05 for > 97% culms), the Weibull’s lower AIC values and near-isometric *G_W_−G_P_* regression (slope’s 95% CI: 0.929−1.039) confirm its superiority for capturing multiplicative growth processes (Weibull, 1951). The observed isometry (intercept ≈ 0, slope ≈ 1) between *G_W_* and *G_P_* is methodologically critical: it demonstrates that the Weibull shape parameter *α* alone can directly quantify inequality without systematic scaling bias. Biologically, this allows efficient prediction of canopy resource allocation asymmetry directly from distribution parameters, bypassing labor-intensive Lorenz curve construction and preserving ecological interpretability of inequality metrics. This approach enables reliable GC prediction from distribution parameters, circumventing labor-intensive Lorenz curve construction. However, generalizability to species with threshold effects (*c* > 0 decoupling GC from skewness; Lian et al., 2024) requires further testing.

The slightly higher pass rate of the Gamma distribution (99%) compared to the Weibull distribution (97.5%) in the K-S tests can be interpreted with an understanding of the purpose and limitations of such hypothesis tests. The K-S test evaluates the null hypothesis that the data are drawn from a specified theoretical distribution. A non-significant result (*p* > 0.05) merely indicates that the distribution cannot be rejected as a plausible model for the data. The K-S test at a significance level of 0.05 is fundamentally a test of minimum adequacy, not a measure of relative model quality or validity. As emphasized by Wasserstein et al. (2019), a *p*-value alone does not measure the size of an effect, the importance of a result, or the probability that a model is correct. It simply quantifies the compatibility between the observed data and a specified statistical model (the null hypothesis). The fact that both distributions were not rejected for the vast majority of culms confirms that both the Gamma and Weibull distributions are generally adequate descriptors of the intra-culm leaf area data within this binary hypothesis-testing paradigm. Model selection, however, requires a different approach. Information criteria such as the AIC are designed for this purpose. AIC estimates the relative quality of statistical models by balancing goodness-of-fit against model complexity, thereby directly addressing trade-offs and guarding against overfitting (Spiess and Neumeyer, 2010). The consistently lower AIC values for the Weibull distribution provide robust evidence that it is a superior model for characterizing intra-culm leaf area distributions, even though the Gamma distribution is also an adequate descriptor. This conclusion aligns with the principles put forward by Wasserstein et al. (2019) to move beyond a dichotomous “significant vs. non-significant” mindset and towards a more nuanced, multi-faceted evaluation of statistical evidence, where continuous measures of model performance (like AIC) are given priority over binary outcomes based on arbitrary *p*-value thresholds.

### 4.3 Limitations and future research directions

We recognize that the exclusive focus on *S. densiflora* limits the direct generalizability of our findings. Future investigations should test this Weibull−Gini approach across a broader phylogenetic spectrum, particularly in other grasses with contrasting life histories and in woody plants where developmental constraints and hydraulic architecture differ substantially from monocots (Küppers, 1989; Doust, 2007). A critical direction involves integrating the effects of abiotic stressors, such as drought, which are known to alter biomass partitioning strategies. Indeed, water scarcity often induces shifts in allometric relationships, potentially amplifying leaf size inequality within a plant as a hydraulic safety strategy. For example, trees under drought stress may produce a greater proportion of smaller leaves to reduce total transpirational area and minimize xylem embolism risk (Brodribb et al., 2020). Applying our protocol in such contexts could determine if the Weibull shape parameter consistently reflects these stress-induced allocation shifts. Moreover, the role of competition, a key driver of architectural variation, warrants explicit investigation. Although our study inferred light competition from vertical foliage arrangement, direct manipulation of planting density or neighbor removal experiments could clarify how resource depletion affects intra-culm leaf area distributions, as studies on woody species demonstrate that branch retention and crown expansion are highly sensitive to neighborhood competition, with branches often exhibiting directional growth to avoid heterospecific neighbors (Sumida et al., 2002). The modular nature of grasses makes them ideal for studying such effects, since resource integration between ramets can buffer or exacerbate inequality, and expanding this approach to large, modular bamboos like *P. edulis* would test the model’s limits under complex, potentially multimodal distributions arising from reiterated branching. The framework also requires testing in species exhibiting threshold effects (location parameter *c* > 0), where the Gini coefficient may diverge from distributional skewness (Lian et al., 2024). In this context, comparative studies with other monocots such as maize could determine whether the Weibull model generalizes across graminoids with differing degrees of apical dominance and branching modularity. The vertical leaf distribution in maize has previously been modeled using simplified bell-shaped functions (Fan et al., 2020). Meanwhile, exploring solitary broadleaved trees such as olive may elucidate how allometric constraints in woody plants influence intra-plant inequality patterns. Olive leaf distribution has been characterized using geometric and image-based methods (Čermák et al., 2007). Ultimately, cross-species comparisons could establish whether a universal scaling relationship exists between the Weibull shape parameter and leaf area inequality. Such comparisons would also clarify whether different plant functional groups converge on distinct strategies quantified by this distribution. These insights would significantly enhance the predictive power of allometric theory in plant ecology.

## 5 Conclusions

This study confirms the two-parameter Weibull distribution as a robust model for quantifying intra-culm inequality in leaf area distribution, validated through rigorous analysis of 9,242 leaves across 121 *Semiarundinaria densiflora* culms. The two-parameter Weibull distribution demonstrated superior performance over the normal, log-normal, and two-parameter Gamma distributions, evidenced by significantly lower AIC values and Kolmogorov-Smirnov goodness-of-fit tests. Crucially, its shape parameter (*α*) enabled precise prediction of observed Gini coefficients derived from Lorenz polygons, with regression slopes (95% CI: 0.929, 1.039) and intercepts (95% CI: −0.006, 0.017) statistically consistent with isometry. This parametric approach provides an efficient alternative to labor-intensive polygon-based GC calculation and captures the multiplicative growth processes inherent in plant development. Biologically, the consistent right-skewed distributions, as indicated by Weibull shape parameters (*α*) of 2.20−3.55, reflect adaptive optimization where apical dominance prioritizes carbon allocation to larger upper leaves for light capture, whereas smaller basal leaves reduce hydraulic risks and self-shading. Notably, pooling leaves across culms invalidated unimodal distributions due to microhabitat-induced multimodality, confirming that intra-culm inequality assessments require organism-level analysis. Our validation of distribution-derived GCs resolves a critical methodological gap in plant allometry by empirically bridging statistical models with ecological inequality metrics. The Weibull−Gini approach, established here for the “woody” grass *S. densiflora*, quantifies intra- culm inequality in leaf area distribution. This approach is particularly suited to plants permitting a practical complete census of leaves. Future tests should therefore target a spectrum of herbaceous plants such as cereals (e.g., maize) and turf grasses (e.g., fescue) with their distinct shoot architectures, and notably extend to woody plant saplings (e.g., *Populus*, *Quercus* and *Salix*), where the approach could be directly applied. In contrast, for mature trees, the effort of measuring every leaf is often prohibitive, posing a significant challenge to direct application. Evaluating the approach across this spectrum of organizational complexity, from simple grasses to complex young trees, can clarify its general utility and establish a broader baseline for comparing intra-plant leaf size variation.

## Data Availability

The original contributions presented in the study are included in the article/**Supplementary Material**. Further inquiries can be directed to the corresponding authors.
